# Strategies to implement and sustain medication use for alcohol and opioid disorders

**DOI:** 10.1186/1940-0640-10-S1-A11

**Published:** 2015-02-20

**Authors:** James H Ford, Raina Croff, Mady Chalk, Kelly Alanis-Hirsch, Kim Johnson, Laura Schmidt, Dennis McCarty

**Affiliations:** 1 Center for Health Systems Research and Analysis, University of Wisconsin-Madison, Madison, WI, 53726, USA; 2 Department of Public Health and Preventive Medicine, Oregon Health & Sciences University, Portland, OR, 97239-3098, USA; 3 Center for Policy Research and Analysis, Treatment Research Institute, Philadelphia, PA, 19106, USA; 4 Department of Psychiatry and Behavioral Sciences, Duke University School of Medicine, Durham, NC, 27710, USA; 5 Center for Health Enhancement System Studies, University of Wisconsin-Madison, Madison, WI, 53726, USA; 6 Philip R. Lee Institute for Health Policy Studies and Department of Anthropology, History and Social Medicine, University of California at San Francisco, CA, 94118, USA

## Background

Medication for treatment of alcohol and opioid use disorders decreases relapse rates and increases long-term recovery. Inclusion of medications in treatment plans, however, may be a complex process for addiction treatment centers. Sustaining use of medications requires additional well-organized and concerted efforts. Qualitative interviews explore implementation and sustainability strategies and barriers encountered by participating providers to promote adoption of medication for opioid- and alcohol-dependent patients.

## Methods

The study included nine intervention sites, four nonintervention sites, and a large commercial health plan. Intervention sites received training, implementation support, and participated in qualitative interviews, while comparison sites only participated in interviews. Qualitative interviews extracted data about strategies utilized to implement and sustain the use of medications in each site.

## Results

Ten specific implementation and sustainability strategies were identified: 1) communicate the benefits and impact of medication to clients; 2) involve staff in the change process to promote buy-in and use of medication to support recovery; 3) involve senior leadership and board support; 4) develop systems to track medication outcomes; 5) integrate medication use into organizational culture and mission; 6) enhance interagency collaboration and payer relationships; 7) dedicate resources to support medication use; 8) develop policies and procedures to support medication use; 9) provide staff training; and 10) identify a sustain champion.

## Conclusions

Like other organizational changes, efforts to implement and sustain changes for the use of medication to support recovery required complex interventions and, in some programs, change in organizational philosophy. Participating programs employed multiple strategies requiring coordination across multiple internal and external stakeholders. They needed staff training, access to prescribers, financing to pay for prescribers, and in some programs, required linkages with pharmaceutical companies. To be successful, treatment providers must devise an effective implementation and sustainability plan.

